# Trajectories of psychological distress and associated factors in patients with postoperative Low Anterior Resection Syndrome: a longitudinal latent class analysis

**DOI:** 10.3389/fpsyg.2026.1919395

**Published:** 2026-08-19

**Authors:** Xiangdong Hu, Qiushi Yin, Mei Cao, Changcui Liang, Hui Yang

**Affiliations:** 1 Department of General Surgery, Lu'an Hospital of Anhui Medical University (Lu'an People's Hospital), Lu'An, China; 2 Graduate College, Wannan Medical University, Wuhu, China; 3 Department of Urology, Affiliated Hospital of West Anhui Health Vocational College, Lu'An, China; 4 Residency Training and Education Department, Lu'an Hospital of Anhui Medical University (Lu'an People's Hospital), Lu'An, China

**Keywords:** colorectal cancer, Latent Growth Mixture Modeling, Low Anterior Resection Syndrome, psychological distress, stigma, survivorship

## Abstract

**Objective:**

To identify distinct trajectories of psychological distress from pre-discharge to 6 months post-discharge in patients with low- and mid-rectal cancer with postoperative LARS and to explore factors associated with different trajectory classes.

**Methods:**

A longitudinal study was conducted among patients with low- and mid-rectal cancer with postoperative LARS recruited from a tertiary hospital in Anhui, China, between March 2024 and June 2025. Psychological distress was assessed using the Kessler Psychological Distress Scale (K10) within 3 days before discharge and at 1, 3, and 6 months after discharge. Of the 205 patients who completed the baseline assessment, 178 completed all follow-up assessments and were included in the final analysis. Latent Growth Mixture Modeling (LGMM) was applied to identify distinct trajectories of psychological distress, and multinomial logistic regression was used to examine factors associated with trajectory class membership.

**Results:**

Overall, psychological distress declined over time; however, substantial heterogeneity in developmental trajectories was observed. Three trajectory classes were identified: (1) mild-high distress with sustained relief (50.0%), (2) moderate-low distress with fluctuating improvement (28.1%), and (3) severe-persistent high distress (21.9%). Primary caregiver, tumor stage, LARS score, and stigma (SIS score) were significantly associated with trajectory class membership (all *p* < 0.05).

**Conclusion:**

Psychological distress among patients with postoperative LARS demonstrates significant heterogeneity over time. Early identification of patients at high risk of persistent distress, particularly those with severe bowel dysfunction, advanced tumor stage, non-family caregivers, and high levels of stigma, is essential. These findings may inform risk stratification and support the development of individualized nursing interventions targeting symptom management, stigma reduction, family support, and psychological adaptation.

## Introduction

Colorectal cancer has become a major public health challenge worldwide. According to global cancer statistics, colorectal cancer ranks among the leading causes of cancer incidence and mortality, with rectal cancer accounting for a substantial proportion of the global disease burden (Bray et al., 2024; Morgan et al., 2023). China contributes nearly 30% of newly diagnosed colorectal cancer cases and related deaths worldwide (National Health Commission of the People's Republic of China Medical Administration Bureau and Chinese Society of Clinical Oncology, 2025), and this burden is expected to increase further due to population aging and lifestyle changes (Xi and Xu, 2021). Among patients with rectal cancer, low- and mid-rectal cancer represent the most common subtypes (Du et al., 2021).

Advances in sphincter-preserving surgical techniques have significantly improved survival outcomes while maintaining anal anatomical integrity (You et al., 2024). However, a considerable proportion of patients develop Low Anterior Resection Syndrome (LARS) after surgery, which has emerged as a major factor affecting postoperative quality of life (Lauwereins et al., 2025; Trenti et al., 2018). LARS is characterized by symptoms such as increased stool frequency, urgency, fecal incontinence, and incomplete evacuation. These symptoms often persist for months or even years, substantially impairing patients’ daily functioning and social participation (Ansar et al., 2024). Previous studies have shown that bowel dysfunction not only causes physical discomfort but also negatively affects psychosocial adjustment and long-term well-being (Stanculea et al., 2025). Many patients reduce outdoor activities or avoid social interactions because of fear of incontinence, resulting in social withdrawal and impaired role functioning (Temple, 2019; Luo et al., 2021).

Psychological distress refers to a state of non-specific emotional suffering that commonly manifests as anxiety, depression, and emotional tension in response to stressful events or health-related challenges (Cheng et al., 2025; Ostovar et al., 2022). It is widely recognized as an important indicator of mental health status. Persistent psychological distress may increase the risk of anxiety and depressive disorders and adversely affect overall health outcomes (Ridner, 2004). Existing evidence suggests a close relationship between bowel dysfunction and psychological distress among patients undergoing rectal cancer surgery. Persistent defecation-related symptoms may lead to embarrassment, body image concerns, and heightened uncertainty in social situations, thereby increasing psychological burden (Rethy et al., 2025). Furthermore, bowel dysfunction may contribute to stigma-related experiences, which can further compromise psychological adaptation and social functioning during cancer survivorship.

The Common-Sense Model of Self-Regulation (CSM) provides a useful theoretical framework for understanding psychological responses to illness (Leventhal et al., 2016). According to this model, individuals develop cognitive and emotional representations of their illness, which subsequently influence coping behaviors and health outcomes. The CSM has been widely applied in studies investigating psychological adaptation across various chronic disease populations (Hayes et al., 2020). In patients with low- and mid-rectal cancer, persistent LARS symptoms may act as ongoing disease-related stressors that influence psychological adaptation during recovery. The CSM provides a useful framework for understanding how patients may respond differently to these challenges over time. Importantly, psychological responses are dynamic rather than static, suggesting that patients may experience different patterns of psychological adaptation throughout cancer survivorship.

Despite growing interest in the psychological consequences of LARS, most previous studies have employed cross-sectional designs that assess psychological status at a single time point (Wang and Pan, 2025; Wang et al., 2024). Such approaches are unable to capture the dynamic evolution of psychological distress throughout postoperative recovery. Furthermore, conventional variable-centered analyses typically assume homogeneous developmental patterns across individuals, potentially overlooking meaningful heterogeneity within the population. In reality, patients may experience markedly different trajectories of psychological distress following surgery.

Latent Growth Mixture Modeling (LGMM) is a person-centered longitudinal approach that enables the identification of distinct developmental trajectories within heterogeneous populations (Mesidor et al., 2023). By uncovering latent subgroups with different patterns of change over time, LGMM provides a more comprehensive understanding of individual differences in psychological adaptation. However, few studies have explored the heterogeneity of psychological distress trajectories among patients with low- and mid-rectal cancer with postoperative LARS using LGMM. Understanding these trajectories and their associated factors, particularly symptom burden and stigma, may facilitate the early identification of patients at high risk of persistent psychological distress and support the development of targeted nursing interventions.

Therefore, this study aimed to identify distinct trajectories of psychological distress from pre-discharge to 6 months after discharge among patients with low- and mid-rectal cancer with postoperative LARS and to explore factors associated with trajectory class membership. The findings may inform individualized psychological care and postoperative rehabilitation strategies.

## Methods

Participants were recruited using convenience sampling from the Departments of Anorectal Surgery and General Surgery at a tertiary Grade A hospital in Anhui Province, China, between March 2024 and June 2025. Eligible participants were patients with low- and mid-rectal cancer with postoperative Low Anterior Resection Syndrome (LARS).

The inclusion criteria were as follows: (1) aged 18 years or older; (2) pathologically confirmed low- or mid-rectal cancer (tumor located within 10 cm of the dentate line) with postoperative LARS identified using the validated LARS score; (3) ability to understand and complete the study procedures, with written informed consent provided. The exclusion criteria were: (1) a history of colorectal malignancy or other concurrent malignant tumors; (2) intestinal obstruction, perforation, hemorrhage, or other conditions requiring emergency surgery; and (3) a documented history of severe psychiatric disorders requiring psychiatric treatment or hospitalization, as identified from the patients’ medical records. Participants were withdrawn if they had incomplete baseline data, voluntarily withdrew from the study, or were unable to complete the follow-up assessments.

According to the principle that the sample size for multivariable regression analysis should be 5–10 times the number of independent variables (Ni et al., 2010), the minimum sample size required for this study was estimated to be 70–140 participants. Considering an anticipated attrition rate of 20%, the adjusted minimum sample size was calculated using the formula *n*/(1−*d*), where n represents the minimum required sample size and d represents the expected attrition rate. Therefore, at least 175 participants were required (140/0.80). A total of 205 eligible patients were recruited at baseline. During follow-up, 7 participants were lost at 1 month, 8 at 3 months, and 12 at 6 months. Ultimately, 178 participants completed all follow-up assessments and were included in the final analysis.

The study was approved by the institutional ethics committee (Approval No. 2024LLKS-KY-028). Written informed consent was obtained from all participants prior to enrollment.

## Study instruments

### General information questionnaire

A self-designed general information questionnaire was used to collect participants’ demographic and clinical characteristics, including age, sex, educational level, primary caregiver, medical payment method, body mass index (BMI), tumor stage, metastasis status, surgical approach, treatment modality, and distance of the tumor from the anal verge. These variables were considered potential associated factors of psychological distress trajectory class membership.

### 10-item Kessler Psychological Distress Scale (K10)

Psychological distress was assessed using the 10-item Kessler Psychological Distress Scale (K10), which measures the frequency of non-specific psychological distress symptoms, including anxiety and emotional tension, experienced during the previous 4 weeks (Kessler et al., 2002). The scale was originally developed by Kessler et al. (2002) and subsequently translated and validated in Chinese populations by Zhou et al. (2009). The K10 consists of 10 items rated on a 5-point Likert scale ranging from 1 (“none of the time”) to 5 (“all of the time”). Total scores range from 10 to 50, with higher scores indicating greater psychological distress. Scores of 10–15 indicate no distress, 16–21 mild distress, 22–29 moderate distress, and 30–50 severe distress. In the present study, the Cronbach’s *α* coefficient was 0.898, indicating good internal consistency.

### Low Anterior Resection Syndrome Score (LARS)

The Low Anterior Resection Syndrome Score (LARS) was developed by Emmertsen and Laurberg (2012) to assess bowel dysfunction following rectal cancer surgery. The scale consists of five items evaluating incontinence for flatus, incontinence for liquid stools, frequency, clustering, and urgency. The Chinese version was translated and validated by Cao et al. (2013) and has demonstrated good reliability and validity in Chinese patients with rectal cancer. Yan et al. further confirmed its usefulness in assessing bowel dysfunction severity after sphincter-preserving surgery (Yan et al., 2015). Total scores range from 0 to 42, with lower scores indicating better bowel function. Scores of 0–20 indicate no LARS, 21–29 indicate minor LARS, and scores ≧30 indicate major LARS.

### Social Impact Scale (SIS)

Perceived stigma was assessed using the Social Impact Scale (SIS), originally developed by Fife and Wright (2000). The Chinese version was revised and validated by Shen et al. (2017) and has been applied in patients with low- and mid-rectal cancer with postoperative LARS. The scale contains 20 items across four dimensions: social rejection (7 items), financial insecurity (3 items), internalized shame (3 items), and social isolation (7 items). Items are scored on a Likert scale, with total scores ranging from 20 to 80. Higher scores indicate greater perceived stigma. Scores of 20–39, 40–59, and 60–80 represent low, moderate, and high levels of stigma, respectively. Previous studies demonstrated good reliability and validity, with a Cronbach’s *α* coefficient of 0.916 for the total scale and values exceeding 0.70 for all subscales. In the present study, the Cronbach’s *α* coefficient of the SIS was 0.943, indicating excellent internal consistency.

### Data collection and quality control

Data were collected at four time points: during hospitalization (T0, within 3 days before discharge) and at 1 month (T1), 3 months (T2), and 6 months (T3) after discharge. At baseline (T0), trained research staff conducted face-to-face interviews at the patients’ bedside using standardized instructions to collect demographic information, psychological distress scores, LARS scores, and stigma levels. Clinical information, including tumor stage and available treatment-related information, was extracted from medical records.

Follow-up assessments at T1–T3 were conducted within ± 1 week of each scheduled assessment using a combination of outpatient visits, telephone interviews, and We Chat communication. Follow-up appointments were arranged in advance and adjusted according to participants’ availability. For participants who experienced difficulty completing questionnaires independently, trained research staff read each item verbatim and recorded responses according to the participants’ answers. If participants exhibited significant emotional distress or requested psychological assistance during data collection, they were referred to appropriate healthcare professionals for further evaluation and support according to routine clinical practice.

To minimize attrition and enhance compliance, several strategies were implemented, including scheduling follow-up assessments at convenient times, maintaining regular contact with participants, and utilizing outpatient visits or hospital readmissions whenever possible. All collected data underwent completeness checks, logical consistency verification, and double-entry validation to ensure data quality and accuracy.

### Statistical analysis

Statistical analyses were performed using SPSS version 26.0 and Mplus version 8.3. Categorical variables were described as frequencies and percentages, whereas continuous variables with normal distributions were expressed as mean ± standard deviation (SD). Differences among trajectory classes were examined using the chi-square test for categorical variables and one-way analysis of variance (ANOVA) for continuous variables.

Latent Growth Mixture Modeling (LGMM) was conducted using Mplus 8.3 to identify distinct trajectories of psychological distress among patients with low-and mid-rectal cancer with postoperative LARS. Model selection was based on statistical fit indices, classification accuracy, interpretability, and clinical relevance. The Akaike Information Criterion (AIC), Bayesian Information Criterion (BIC), sample-size adjusted BIC (aBIC), entropy, Lo–Mendell–Rubin adjusted likelihood ratio test (LMRT), and bootstrap likelihood ratio test (BLRT) were used to evaluate model fit. Lower AIC, BIC, and aBIC values indicate better model fit, whereas higher entropy values indicate greater classification accuracy. Significant LMRT and BLRT results (*p* < 0.05) suggest that the model with *k* classes provides a better fit than the model with *k*−1 classes.

Variables with statistical significance in univariate analyses were entered into multinomial logistic regression models to identify factors associated with latent trajectory class membership. Statistical significance was defined as a two-sided *p* value <0.05.

## Results

### Participant flow and follow-up

**Figure 1:**
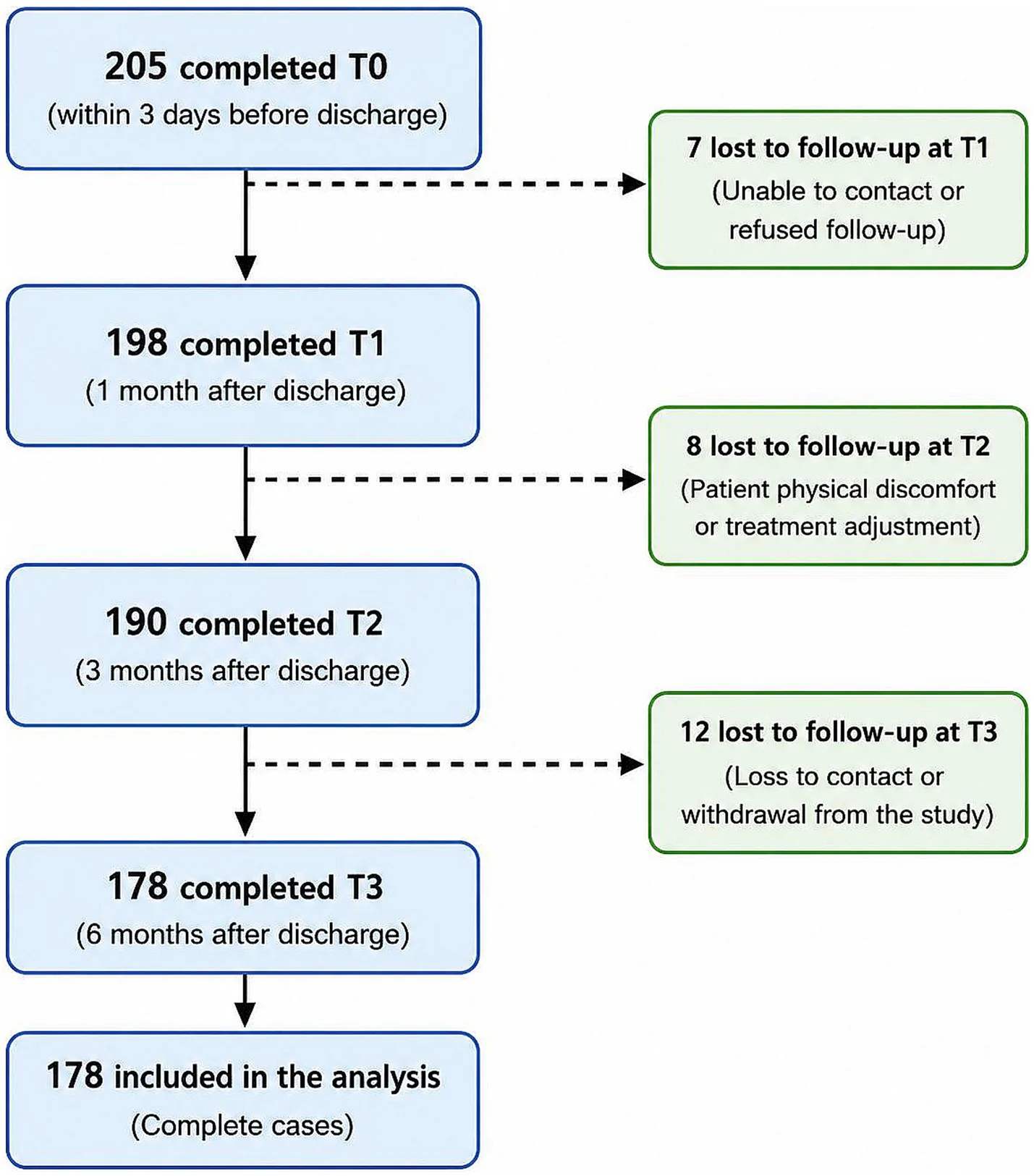
Flow diagram of participant recruitment and follow-up. T0 = baseline; T1 = 1 month; T2 = 3 months; T3 = 6 months after discharge.

A total of 205 patients completed the baseline assessment (T0). During the follow-up period, 7 patients were lost at T1 owing to inability to contact or refusal to continue participation, 8 were lost at T2 because of physical discomfort or treatment adjustments, and 12 were lost at T3 due to loss of contact or withdrawal from the study. Consequently, 178 patients completed all follow-up assessments and were included in the final analysis. The participant flow is presented in Figure 1.

### Baseline characteristics

Baseline characteristics of the participants are presented in Table 1. At baseline, LARS scores ranged from 33 to 40, indicating that all participants met the criteria for major LARS.

**Table 1:** Comparison of baseline characteristics between participants who completed follow-up and those lost to follow-up.

Variables	Category	Completed follow-up (*n* = 178)	Lost to follow-up (*n* = 27)	*χ*^2^/*t*	*p*
Sex, *n* (%)	Male	118 (66.29)	14 (51.85)	2.132	0.195
	Female	60 (33.70)	13 (48.15)		
Age, *n* (%)	<60 years	57 (32.02)	7 (25.93)	0.406	0.657
	≧60 years	121 (67.98)	20 (74.07)		
Educational level, *n* (%)	≤Junior high	110 (61.80)	21 (77.78)	2.595	0.134
	>Junior high	68 (38.20)	6 (22.22)		
BMI, *n* (%)	<24 kg/m^2^	136 (76.40)	19 (70.37)	0.463	0.631
	≧24 kg/m^2^	42 (23.60)	8 (29.63)		
Primary caregiver, *n* (%)	Family member	119 (66.85)	14 (51.85)	2.316	0.136
	Non-family member	59 (33.15)	13 (48.15)		
Tumor stage, *n* (%)	T1	31 (17.42)	0 (0.00)	5.954	0.107
	T2	58 (32.58)	9 (33.33)		
	T3	74 (41.57)	15 (55.56)		
	T4	15 (8.43)	3 (11.11)		
K10 score, mean ± SD		34.11 ± 8.56	33.85 ± 4.99	−0.151	0.880
LARS score, mean ± SD		37.33 ± 1.58	37.19 ± 1.24	−0.528	0.601
SIS score, mean ± SD		67.56 ± 4.03	66.85 ± 4.05	−0.842	0.406

Data are presented as *n* (%) or mean ± SD. BMI, body mass index; K10, 10-item Kessler Psychological Distress Scale; LARS, Low Anterior Resection Syndrome Score; SIS, Social Impact Scale. *χ*^2^ values were used for categorical variables and *t* values for continuous variables.

### Attrition analysis

To assess the potential influence of attrition bias, baseline characteristics were compared between participants who completed all follow-up assessments (*n* = 178) and those who were lost to follow-up (*n* = 27). No significant differences were observed between the two groups with respect to sex, age, educational level, BMI, primary caregiver, tumor stage, baseline psychological distress (K10 score), LARS score, or stigma level (SIS score) (all *p* > 0.05), suggesting that loss to follow-up was unlikely to introduce substantial bias into the study findings (Table 1).

### Changes in psychological distress over time

The mean psychological distress score was 34.11 ± 6.90 at baseline, decreasing to 25.80 ± 10.76, 23.92 ± 10.99, and 22.55 ± 11.53 at 1, 3, and 6 months after discharge, respectively. Overall, psychological distress declined significantly over time.

Repeated-measures analysis of variance revealed a significant effect of time on psychological distress scores (*F* = 85.629, *p* < 0.001), indicating significant differences across the four assessment time points.

### Identification of latent trajectory classes of psychological distress

Latent Growth Mixture Modeling (LGMM) was conducted using psychological distress scores measured at four time points (baseline, 1 month, 3 months, and 6 months after discharge) as observed indicators. Models with one to five latent classes were estimated and compared using the Akaike Information Criterion (AIC), Bayesian Information Criterion (BIC), sample-size adjusted BIC (aBIC), entropy, Lo–Mendell–Rubin adjusted likelihood ratio test (LMRT), and bootstrap likelihood ratio test (BLRT). The model fit indices are presented in Table 2.

**Table 2:** Model fit statistics for latent growth mixture models of psychological distress trajectories.

Number of classes	AIC	BIC	aBIC	Entropy	LMRT *p*-value	BLRT *p*-value	Class proportions
1	4557.148	4585.784	4557.282	/	/	/	/
2	4424.152	4462.333	4424.331	0.983	<0.001	<0.001	0.281/0.719
3	4146.063	4193.790	4146.287	0.997	<0.001	<0.001	0.219/0.500/0.281
4	4104.360	4161.632	4104.628	0.947	0.032	0.026	0.135/0.365/0.281/0.219
5	4073.127	4139.945	4073.440	0.924	0.205	0.186	0.141/0.281/0.258/0.219/0.101

AIC, Akaike Information Criterion; BIC, Bayesian Information Criterion; aBIC, sample-size adjusted Bayesian Information Criterion; LMRT, Lo–Mendell–Rubin adjusted likelihood ratio test; BLRT, bootstrap likelihood ratio test. Lower AIC, BIC, and aBIC values indicate better model fit. Higher entropy values indicate greater classification accuracy.

As the number of classes increased, AIC, BIC, and aBIC values decreased. Although the five-class model showed the lowest information criteria, its LMRT and BLRT were non-significant, and the additional classes lacked clinical interpretability. The four-class model showed good classification accuracy (entropy = 0.947) with significant LMRT and BLRT results, but did not provide meaningful improvement. The three-class model demonstrated excellent classification accuracy (entropy = 0.997), significant LMRT and BLRT results (both *p* < 0.001), and adequate class proportions (>10%). Therefore, the three-class model was selected as the optimal solution.

### Identification and characterization of psychological distress trajectories

**Figure 2:**
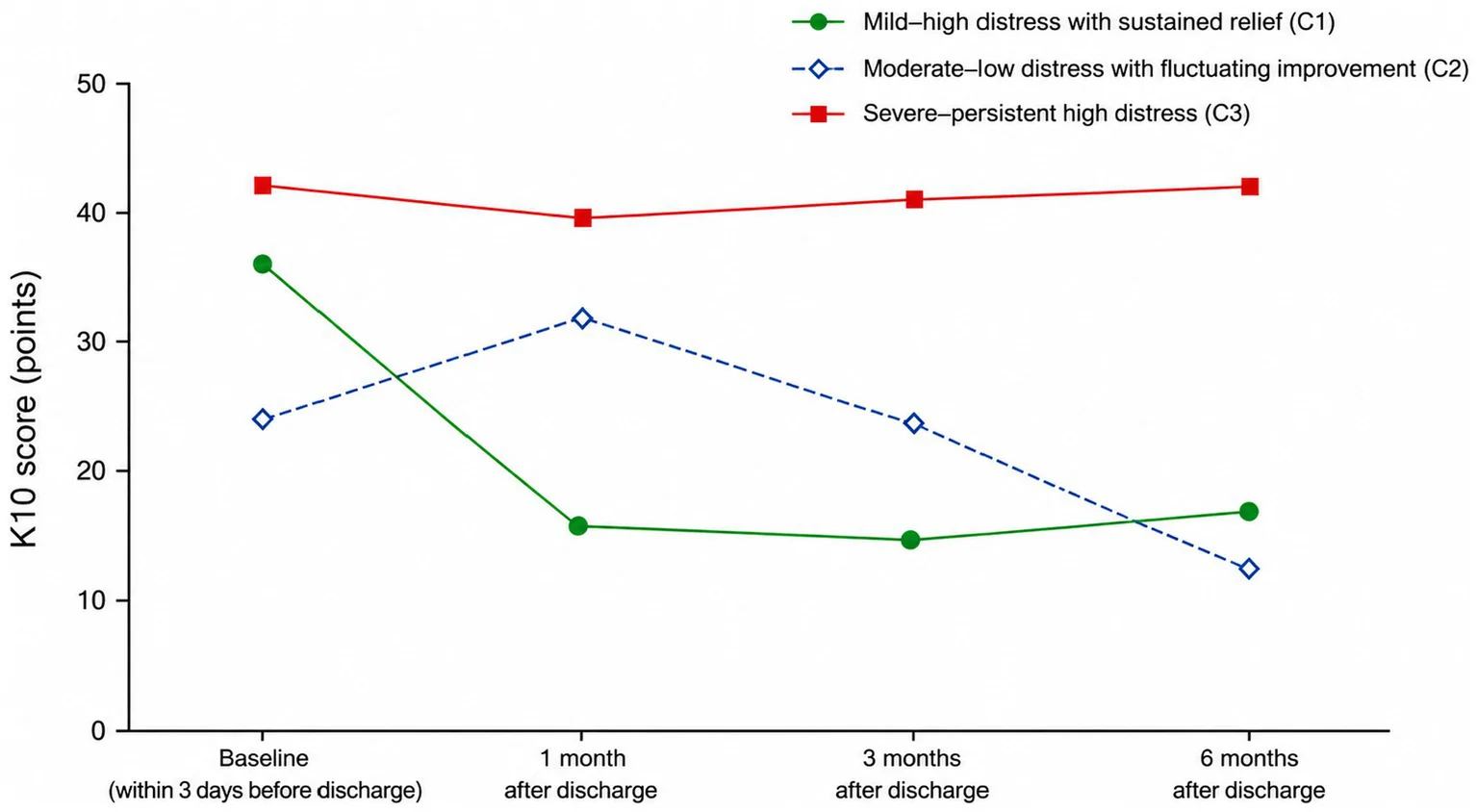
Trajectories of psychological distress identified by latent growth mixture modeling among patients with low- and mid-rectal cancer complicated by LARS. C1 = mild-high distress with sustained relief (50.0%, *n* = 89); C2 = moderate-low distress with fluctuating improvement (28.1%, *n* = 50); C3 = severe-persistent high distress (21.9%, *n* = 39).

Based on the optimal three-class LGMM solution, three distinct trajectories of psychological distress were identified among patients with low- and mid-rectal cancer complicated by LARS (Figure 2).

Class 1 comprised 50.0% of the sample (*n* = 89). Participants in this class exhibited relatively high levels of psychological distress at baseline, followed by a rapid decline during the early recovery period and subsequent stabilization at low levels of distress. This trajectory was therefore labeled the “mild-high distress with sustained relief” group.

Class 2 accounted for 28.1% of the sample (*n* = 50). Participants in this class showed moderate levels of psychological distress at baseline, experienced a transient increase in distress at 1 month after discharge, and then demonstrated a gradual decline over the subsequent follow-up period. This trajectory was labeled the “moderate-low distress with fluctuating improvement” group.

Class 3 represented 21.9% of the sample (*n* = 39). Participants in this class reported persistently high levels of psychological distress throughout the study period, with only minimal changes over time. Accordingly, this trajectory was labeled the “severe-persistent high distress” group.

### Growth parameters of identified psychological distress trajectories

The estimated growth parameters of the three trajectory classes, including the intercept and linear slope, are presented in Table 3. The intercept and slope values reflected the initial levels and longitudinal change patterns of psychological distress, respectively, across the identified trajectory groups.

**Table 3:** Estimated growth parameters of psychological distress trajectories identified by latent growth mixture modeling.

Trajectory class	Intercept	Slope
C1: mild–high distress with sustained relief	33.264	−4.020
C2: moderate–low distress with fluctuating improvement	11.506	−0.476
C3: severe–persistent high distress	44.868	−2.571

Intercept represents the estimated initial level of psychological distress, and slope represents the estimated linear rate of change over time.

### Univariate analysis of factors associated with trajectory class membership

Univariate analyses showed significant differences among the three trajectory classes with respect to educational level, personal income, primary caregiver, tumor stage, LARS score, and stigma (SIS score) (all *p* < 0.05). No significant differences were observed for the remaining variables (all *p* > 0.05). Detailed results are presented in Table 4.

**Table 4:** Univariate analysis of factors associated with psychological distress trajectory classes among patients with low- and mid-rectal cancer complicated by LARS.

Variable	Category	Class 1 (*n* = 89)	Class 2 (*n* = 50)	Class 3 (*n* = 39)	*χ*^2^/*F*	*p*
Gender, *n* (%)	Male	58 (65.17)	31 (62.00)	29 (74.36)	1.598	0.450
	Female	31 (34.83)	19 (38.00)	10 (25.64)		
Age, *n* (%)	<60 years	28 (31.46)	19 (38.00)	12 (30.77)	1.563	0.458
	≥60 years	61 (68.54)	31 (62.00)	27 (69.23)		
Marital status, *n* (%)	Unmarried	11 (12.36)	5 (10.00)	3 (7.69)	0.653	0.722
	Married	78 (87.64)	45 (90.00)	36 (92.31)		
Residence, *n* (%)	Urban	36 (40.45)	12 (24.00)	16 (41.03)	4.319	0.115
	Rural	53 (59.55)	38 (76.00)	23 (58.97)		
Education, *n* (%)	Junior or below	57 (64.04)	23 (46.00)	30 (76.92)	9.255	0.010
	Senior or above	32 (35.96)	27 (54.00)	9 (23.08)		
Income, *n* (%)	<3,000 RMB	15 (16.85)	8 (16.00)	9 (23.08)	16.60	0.002
	3,000–5,000 RMB	62 (69.66)	22 (44.00)	25 (64.10)		
	>5,000 RMB	12 (13.48)	20 (40.00)	5 (12.82)		
Caregiver, *n* (%)	Family	54 (60.67)	43 (86.00)	22 (56.41)	11.725	0.003
	Non-family	35 (39.33)	7 (14.00)	17 (43.59)		
BMI, *n* (%)	<24	65 (73.03)	40 (80.00)	31 (79.49)	1.125	0.570
	≥24	24 (26.97)	10 (20.00)	8 (20.51)		
Tumor stage, *n* (%)	T1	23 (25.84)	4 (8.00)	4 (10.26)	23.698	0.001
	T2	36 (40.45)	9 (18.00)	13 (33.33)		
	T3	24 (26.97)	32 (64.00)	18 (46.15)		
	T4	6 (6.74)	5 (10.00)	4 (10.26)		
Metastasis, *n* (%)	Yes	15 (16.85)	8 (16.00)	3 (7.69)	1.933	0.380
	No	74 (83.15)	42 (84.00)	36 (92.31)		
Chronic disease, *n* (%)	Yes	68 (76.40)	39 (78.00)	33 (84.62)	1.106	0.575
	No	21 (23.60)	11 (22.00)	6 (15.38)		
Distance, *n* (%)	<5 cm	16 (17.98)	8 (16.00)	9 (23.08)	0.764	0.683
	5–10 cm	73 (82.02)	42 (84.00)	30 (76.92)		
LARS score, Mean ± SD		22.35 ± 2.89	19.75 ± 3.82	28.28 ± 5.88	52.252	<0.001
SIS score, Mean ± SD		52.58 ± 11.84	39.28 ± 14.89	63.93 ± 17.16	34.57	<0.001

Data are presented as *n* (%) or mean ± SD. BMI, body mass index; LARS, Low Anterior Resection Syndrome Score; SIS, Social Impact Scale. *χ*^2^ tests were used for categorical variables, and one-way analysis of variance (ANOVA) was used for continuous variables. *p* < 0.05 was considered statistically significant.

### Multinomial logistic regression analysis of factors associated with psychological distress trajectory class membership

Variables that were statistically significant in the univariate analyses were entered into a multinomial logistic regression model. Class 3 (severe-persistent high distress group) was used as the reference category.

The results showed that primary caregiver, LARS score, and stigma (SIS score) were significant factors distinguishing Class 2 (moderate-low distress with fluctuating improvement) from Class 3 (all *p* < 0.05). In addition, tumor stage, LARS score, and SIS score were significant factors distinguishing Class 1 (mild-high distress with sustained relief) from Class 3 (all *p* < 0.05).

Notably, both LARS score and SIS score were significantly associated with trajectory class membership across comparisons, suggesting that bowel dysfunction severity and perceived stigma may play important roles in the development of psychological distress trajectories. Detailed results are presented in Table 5.

**Table 5:** Multinomial logistic regression analysis of factors associated with psychological distress trajectory class membership among patients with low- and mid-rectal cancer complicated by LARS.

Comparison	Predictor	*β*	SE	Wald *χ*^2^	*p*	OR (95%CI)
Class 1 vs. 3	Tumor stage	−1.61	0.80	−2.02	0.044	0.20 (0.04–0.95)
	LARS score	−0.31	0.06	−4.84	<0.001	0.74 (0.65–0.83)
	SIS score	−0.08	0.02	−3.78	<0.001	0.92 (0.88–0.96)
Class 2 vs. 3	Primary caregiver	2.05	0.93	2.20	0.028	7.80 (1.25–48.65)
	LARS score	0.24	0.06	3.82	<0.001	1.27 (1.12–1.44)
	SIS score	0.07	0.02	3.79	<0.001	1.08 (1.04–1.12)

Class 3 (severe-persistent high distress group) was specified as the reference category in the multinomial logistic regression model. *β,* regression coefficient; SE, standard error; Wald *χ*^2^, Wald chi-square statistic; OR, odds ratio; CI, confidence interval; LARS, Low Anterior Resection Syndrome Score; SIS, Social Impact Scale. Statistical significance was defined as *p* < 0.05.

## Discussion

### Heterogeneous trajectories of psychological distress among patients with low- and mid-rectal cancer complicated by LARS

Using latent growth mixture modeling (LGMM), this study identified three distinct trajectories of psychological distress among patients with low- and mid-rectal cancer complicated by LARS during the first 6 months after discharge. Unlike conventional variable-centered approaches, LGMM enabled the identification of heterogeneous developmental patterns at the individual level, providing a more comprehensive understanding of postoperative psychological adaptation. These findings indicate that psychological recovery following rectal cancer surgery is not uniform but characterized by substantial heterogeneity, which is generally consistent with previous longitudinal studies among colorectal cancer survivors (Qaderi et al., 2022).

The largest subgroup demonstrated initially elevated psychological distress followed by sustained improvement over time, suggesting that many patients gradually adapt to postoperative challenges during recovery. Improvements in physical function, enhanced self-management ability, stabilization of illness perceptions, and increasing social support may collectively facilitate psychological recovery. This pattern is consistent with the Common-Sense Model of Self-Regulation (CSM), which proposes that illness perceptions and coping responses evolve throughout the recovery process, thereby influencing emotional adaptation and health outcomes (Leventhal et al., 2016; Hayes et al., 2020).

A second subgroup experienced a transient increase in psychological distress during the first month after discharge before showing gradual improvement. This trajectory may reflect the transition from hospital-based care to home-based self-management. During this period, professional medical support decreases while patients assume greater responsibility for symptom management. Difficulties in controlling bowel symptoms, uncertainty regarding recovery, and challenges in daily functioning may temporarily intensify psychological distress (Sharp et al., 2025; Varghese et al., 2022). These findings suggest that the first month after discharge represents a vulnerable period during which enhanced psychological monitoring and supportive nursing interventions may be particularly beneficial.

Approximately one-fifth of patients remained in the severe-persistent high distress trajectory throughout the follow-up period. These patients appeared to experience inadequate psychological adaptation and may have been exposed to cumulative risk factors, including severe bowel dysfunction, high levels of stigma, and limited social support. Persistent psychological distress may negatively influence treatment adherence, rehabilitation outcomes, and long-term quality of life among colorectal cancer survivors (Zhao et al., 2013; Hu et al., 2025). Therefore, early identification of patients at risk of persistent distress is essential for implementing timely and individualized psycho-oncological interventions.

### Factors influencing psychological distress trajectories among patients with low- and mid-rectal cancer complicated by LARS

Multinomial logistic regression analysis demonstrated that primary caregiver, tumor stage, LARS severity, and stigma were significant predictors of psychological distress trajectory membership.

### Family caregivers promote more favorable psychological adaptation

The present study found that patients whose primary caregivers were family members were less likely to belong to the severe-persistent high distress trajectory than those cared for by non-family caregivers. This finding suggests that family caregiving may reduce the likelihood of persistent psychological distress by providing continuous emotional support, practical assistance, and reassurance throughout postoperative recovery. Consistent with previous studies (Xia et al., 2025; Averyt and Nishimoto, 2014), family caregivers can reduce uncertainty, enhance patients’ sense of security, and facilitate psychological adaptation. In addition, supportive family relationships may help address unmet supportive care needs and improve adjustment during cancer survivorship (Qiu et al., 2025).

These findings underscore the importance of family-centered care. Healthcare professionals should actively involve family caregivers in postoperative rehabilitation by providing education on disease management, symptom monitoring, rehabilitation assistance, and caregiver self-care. Strengthening caregivers’ competence and psychological support skills may contribute to a more supportive home environment and ultimately improve patients’ psychological well-being.

### Advanced tumor stage increases the risk of persistent psychological distress

Tumor stage was identified as an important factor distinguishing the severe-persistent high distress trajectory from the mild-high distress with sustained relief trajectory. Patients with more advanced disease were more likely to experience persistent psychological distress, indicating that disease severity plays an important role in psychological adaptation. Similar findings have been reported in previous studies involving cancer survivors (Averyt and Nishimoto, 2014; Qiu et al., 2025).

Patients with advanced-stage disease often experience greater symptom burden, more intensive treatment demands, increased uncertainty regarding prognosis, and prolonged treatment-related adverse effects. Moreover, advanced disease may increase illness uncertainty and perceived threat, both of which are recognized determinants of psychological distress. Earlier psychosocial assessment, continuous symptom management, and individualized psychological support may therefore help improve coping capacity and reduce psychological burden in this population.

### Greater bowel dysfunction severity is associated with worse psychological outcomes

The present study demonstrated that LARS severity was a significant predictor of psychological distress trajectories. Patients with more severe bowel dysfunction were more likely to experience persistent psychological distress, consistent with previous studies showing that severe LARS substantially impairs quality of life and psychological well-being after rectal cancer surgery (Al Rashid et al., 2022; Zarghani et al., 2025).

Symptoms such as urgency, fecal incontinence, clustering, and increased bowel frequency can substantially interfere with daily activities and social participation. These limitations may negatively affect body image, self-esteem, and self-efficacy while simultaneously increasing social anxiety and emotional burden (Al Rashid et al., 2022; Asefa et al., 2025). Persistent bowel dysfunction may also impair sleep quality, reduce confidence in symptom control, and gradually deplete psychological coping resources, thereby reinforcing psychological distress (Zarghani et al., 2025). From a psycho-oncological perspective, effective management of bowel dysfunction should be regarded not only as a strategy for functional rehabilitation but also as an integral component of psychological care. Interventions including pelvic floor rehabilitation, individualized dietary counseling, pharmacological treatment, and bowel training may therefore improve both physical and psychological outcomes.

### Higher stigma is associated with more severe psychological distress

Stigma emerged as another important predictor of psychological distress trajectories. Patients with higher levels of stigma were more likely to belong to the severe-persistent high distress trajectory, indicating that stigma plays a critical role in the development and maintenance of psychological distress among patients with LARS.

Patients experiencing stigma may internalize negative perceptions regarding bowel dysfunction and cancer, leading to feelings of shame, self-blame, and social withdrawal (Mi et al., 2025). Emerging evidence suggests that stigma is a common yet frequently overlooked experience among colorectal cancer survivors and may substantially impair psychosocial well-being (Zivanov et al., 2025). In addition, concerns regarding social rejection may discourage patients from seeking professional support or discussing bowel-related problems, thereby delaying effective symptom management.

Importantly, stigma does not operate independently. In the present study, stigma was positively associated with LARS severity, which is consistent with previous findings (Feng, 2025). Severe bowel dysfunction may increase embarrassment and impair social functioning, thereby intensifying stigma. Conversely, heightened stigma may reduce help-seeking behaviors and delay symptom management, creating a vicious cycle between symptom burden and psychological distress (Løvall et al., 2024). Routine assessment of stigma should therefore be incorporated into postoperative follow-up, particularly for patients with severe bowel dysfunction and limited social support. Integrating stigma-reduction interventions with symptom management strategies may facilitate psychological adaptation and improve rehabilitation outcomes.

### Clinical implications

From a psycho-oncological perspective, psychological distress among patients with LARS should not be viewed solely as an emotional consequence of cancer treatment but as a multidimensional outcome closely related to bowel dysfunction, stigma, family support, and survivorship experiences. These findings support the implementation of risk-stratified survivorship care that integrates bowel symptom management, psychological support, family involvement, and stigma-reduction strategies. Early psychological screening may facilitate timely identification of patients at high risk of persistent distress and guide the development of individualized nursing interventions throughout postoperative rehabilitation.

## Conclusion

This study identified three distinct trajectories of psychological distress among patients with low- and mid-rectal cancer complicated by Low Anterior Resection Syndrome during the first 6 months after discharge, demonstrating substantial heterogeneity in postoperative psychological adaptation. Primary caregiver, tumor stage, LARS severity, and stigma were independently associated with trajectory class membership. These findings support early risk stratification and individualized psycho-oncological nursing interventions to promote psychological adaptation and optimize survivorship care.

### Limitations and future directions

Several limitations should be acknowledged. First, this study was conducted in a single tertiary hospital in China using convenience sampling, and the follow-up period was limited to 6 months after discharge. These factors may restrict the generalizability of the findings and the evaluation of long-term psychological distress trajectories. Future multicenter studies with larger and more diverse samples and extended follow-up periods are needed to validate the identified trajectories.

Second, although demographic, clinical, and psychosocial factors were considered, some potentially important variables, including neoadjuvant/adjuvant chemoradiotherapy, stoma status, postoperative complications, coping strategies, illness perceptions, and psychological interventions, were not included in the current model. The absence of these variables may have resulted in residual confounding, and future studies should incorporate more comprehensive clinical and psychosocial assessments.

Third, although the sample size was adequate for the present LGMM analysis, the initial estimation was based on the number of variables planned for regression analysis rather than longitudinal model-specific power calculations incorporating effect size, repeated measurements, and within-subject correlation. Future research should adopt sample size estimation approaches specifically designed for longitudinal trajectory models.

Finally, although no significant baseline differences were observed between participants with complete and incomplete follow-up data, complete-case analysis was applied in the present study. Future studies should consider advanced missing-data approaches, such as full information maximum likelihood, to reduce potential bias. In addition, the K10 total score reflects nonspecific psychological distress and does not distinguish anxiety and depressive symptoms; future studies should explore these dimensions separately to provide more targeted clinical insights.

## Data Availability

The raw data supporting the conclusions of this article will be made available by the authors, without undue reservation.

## References

[ref26] Al Rashid F Liberman AS Charlebois P Stein B Feldman LS Fiore JF Jr . (2022). The impact of bowel dysfunction on health-related quality of life after rectal cancer surgery: a systematic review. Tech Coloproctol. (2022). 26, 515–527. doi: 10.1007/S10151-022-02594-0 35239096

[ref1] Ansar M. Boddeti S. Noor K. Malireddi A. Abera M. Suresh S. B. . (2024). A systematic review of comparative effectiveness of interventions for low anterior resection syndrome: impacts on bowel function and quality of life. Cureus. 16:e72772. doi: 10.7759/cureus.72772, 39483606 PMC11527395

[ref2] Asefa T. Endale H. T. Getnet M. Aragie H. Negash H. K. Gela Y. Y. . (2025). The impact of ostomy on colorectal cancer patients and caregivers: a qualitative study. Support Care Cancer 33:1030. doi: 10.1007/s00520-025-10075-x, 41204028 PMC12594726

[ref3] Averyt J. C. Nishimoto P. W. (2014). Psychosocial issues in colorectal cancer survivorship: the top ten questions patients may not be asking. J Gastrointest Oncol. 5, 395–400. 25276412 10.3978/j.issn.2078-6891.2014.058 PMC4173044

[ref4] Bray F. Laversanne M. Sung H. Ferlay J. Siegel R. L. Soerjomataram I. . (2024). Global cancer statistics 2022: GLOBOCAN estimates of incidence and mortality worldwide for 36 cancers in 185 countries. CA Cancer J. Clin. 74, 229–263. doi: 10.3322/caac.21834, 38572751

[ref5] Cao L. Y. Wei L. Wang C. M. (2013). Translation and validation of the Chinese version of the low anterior resection syndrome score. Chin. J. Pract. Nurs. 29, 69–72.

[ref6] Cheng C. F. Yun J. Zhou Z. Q. Wang J. L. (2025). Current status and influencing factors of psychological distress among perimenopausal nurses. Mil. Nurs. 42, 27–30.

[ref7] Du X. H. Feng B. Han J. G. Li X. X. Liu F. L. Liu S. . (2021). Chinese expert consensus on digestive tract reconstruction in surgery for mid-low rectal cancer (2021 edition). Chin. J. Pract. Surg. 41, 1081–1089.

[ref8] Emmertsen K. J. Laurberg S. (2012). Low anterior resection syndrome score: development and validation of a symptom-based scoring system for bowel dysfunction after low anterior resection for rectal cancer. Ann. Surg. 255, 922–928., 22504191 10.1097/SLA.0b013e31824f1c21

[ref9] Feng Q. (2025) The Relationship between low anterior resection syndrome score and quality of life in patients after sphincter-preserving surgery for rectal cancer: the mediating role of experiential avoidance and social alienation. (dissertation). Anhui Medical University, Hefei, China

[ref10] Fife B. L. Wright E. R. (2000). The dimensionality of stigma: a comparison of its impact on the self of persons with HIV/AIDS and cancer. J. Health Soc. Behav. 41, 50–67. doi: 10.2307/2676360, 10750322

[ref11] Hayes B. Moller S. Wilding H. Burgell R. Apputhurai P. Knowles S. R. (2020). Application of the common sense model in inflammatory bowel disease: a systematic review. J. Psychosom. Res. 139:110283. doi: 10.1016/j.jpsychores.2020.110283, 33161175

[ref12] Hu X. Zhang Q. Wang H. Tan J. Geng Q. Ma H. . (2025). Psychological distress among colorectal cancer patient-caregiver dyads: influencing factors and dyadic intervention preferences—a mixed methods study. Psychooncology 34:e70287. doi: 10.1002/pon.70287, 40973983

[ref13] Kessler R. C. Andrews G. Colpe L. J. Hiripi E. Mroczek D. K. Normand S. L. . (2002). Short screening scales to monitor population prevalences and trends in non-specific psychological distress. Psychol. Med. 32, 959–976. doi: 10.1017/S0033291702006074, 12214795

[ref14] Lauwereins L. D’Hoore A. Coeckelberghs E. Fieuws S. Wolthuis A. Bislenghi G. . (2025). A 2-year prospective study on the evolution of low anterior resection syndrome (LARS) following rectal cancer surgery. Int. J. Color. Dis. 40:220. doi: 10.1007/s00384-025-05009-2 PMC12546544 41125949

[ref15] Leventhal H. Phillips L. A. Burns E. (2016). The common-sense model of self-regulation (CSM): a dynamic framework for understanding illness self-management. J. Behav. Med. 39, 935–946. doi: 10.1007/s10865-016-9782-2, 27515801

[ref16] Løvall C. Mjelde L. M. E. Eide L. S. P. Reime M. H. (2024). Patients’ experiences of living with low anterior resection syndrome three to six months after colorectal cancer surgery: a phenomenological study. PLoS One 19:e0305212. doi: 10.1371/journal.pone.0305212, 38985702 PMC11236105

[ref17] Luo B. J. Yang X. Chen C. Y. Zheng M. C. (2021). Current status and correlation between defecation function and quality of life in patients undergoing sphincter-preserving surgery for mid-low rectal cancer. J. Nurses Train. 36, 2209–2213.

[ref18] Mesidor M. Sirois C. Simard M. Talbot D. (2023). A bootstrap approach for evaluating uncertainty in the number of groups identified by latent class growth models. Am. J. Epidemiol. 192, 1896–1903. doi: 10.1093/aje/kwad148, 37386696

[ref19] Mi B. Jin Y. Zheng M. Cheng H. Zhang J. (2025). Stigma, colorectal cancer knowledge and self-efficacy among colorectal cancer survivors: a cross-sectional study based on random forest analysis. Eur. J. Oncol. Nurs. 76:102858. doi: 10.1016/j.ejon.2025.102858, 40086202

[ref20] Morgan E. Arnold M. Gini A. Lorenzoni V. Cabasag C. J. Laversanne M. . (2023). Global burden of colorectal cancer in 2020 and 2040: incidence and mortality estimates from GLOBOCAN. Gut 72, 338–344. doi: 10.1136/gutjnl-2022-327736, 36604116

[ref21] National Health Commission of the People's Republic of China Medical Administration Bureau and Chinese Society of Clinical Oncology (2025). Chinese protocol for diagnosis and treatment of colorectal cancer (2025 edition) (concise version). Chin. J. Pract. Surg. 45, 1353–1359.

[ref22] Ni P. Chen J. L. Liu N. (2010). Sample size estimation for quantitative research in nursing studies. Chin. J. Nurs. 45, 378–380.

[ref23] Ostovar S. Modarresi Chahardehi A. Mohd Hashim I. H. Othman A. Kruk J. Griffiths M. D. (2022). Prevalence of psychological distress among cancer patients in southeast Asian countries: a systematic review. Eur J Cancer Care (Engl). 31:e13669. doi: 10.1111/ecc.13669, 35934684 PMC9786346

[ref24] Qaderi S. van der Heijden J. Verhoeven R. de Wilt J. Custers J. (2022). Trajectories of health-related quality of life and psychological distress in patients with colorectal cancer: a population-based study. Eur. J. Surg. Oncol. 48:e110. doi: 10.1016/j.ejso.2021.12.180 34666216

[ref25] Qiu X. Mao J. Wang C. Yang X. Zhao J. Li Q. (2025). Interdependence relationships between unmet supportive care needs and its influencing factors in couples coping with colorectal cancer: a cross-sectional study. Support Care Cancer 33:297. doi: 10.1007/S00520-025-09350-8, 40102327

[ref27] Rethy B. Schandl A. Nordenvall C. Palmer G. J. Bergström C. Williamson M. . (2025). The patient perspective on transanal irrigation treatment for low anterior resection syndrome after rectal cancer surgery: a qualitative and quantitative study. BMC Gastroenterol. 25:64. doi: 10.1186/s12876-025-03633-4, 39920596 PMC11804026

[ref28] Ridner S. H. (2004). Psychological distress: concept analysis. J. Adv. Nurs. 45, 536–545. doi: 10.1046/j.1365-2648.2003.02938.x, 15009358

[ref29] Sharp G. Findlay N. Clark D. Hong J. (2025). Systematic review of the management options available for low anterior resection syndrome (LARS). Tech. Coloproctol. 29:58. doi: 10.1007/s10151-024-03090-3, 39903381

[ref30] Shen Q. Z. Mou S. Y. Wang X. H. Liu Y. Reliability and validity evaluation of the Chinese version of the Social Impact Scale applied to stigma in patients with enterostomy. J Chongqing Med Univ. (2017) 42:1188–1192.

[ref31] Stanculea F. Ungureanu C. Roca D. Ginghina O. Mihailov R. Grama F. (2025). The impact of low anterior resection syndrome (LARS) on the quality of life in rectal cancer survivors: a narrative review. Maedica (Bucur). 20, 56–64. doi: 10.26574/maedica.2025.20.1.56 40677670 PMC12123494

[ref32] Temple L. K. F. (2019). Expert commentary on low anterior resection syndrome. Dis. Colon Rectum 62, 1423–1424. doi: 10.1097/DCR.0000000000001531, 31725579

[ref33] Trenti L. Galvez A. Biondo S. Solis A. Vallribera-Valls F. Espin-Basany E. . (2018). Quality of life and anterior resection syndrome after surgery for mid to low rectal cancer: a cross-sectional study. Eur. J. Surg. Oncol. 44, 1031–1039. doi: 10.1016/j.ejso.2018.03.025, 29665980

[ref34] Varghese C. Wells C. I. O’Grady G. Christensen P. Bissett I. P. Keane C. (2022). The longitudinal course of low anterior resection syndrome: an individual patient meta-analysis. Ann. Surg. 276, 46–54. doi: 10.1097/SLA.0000000000005423, 35185131

[ref35] Wang G. Pan S. (2025). Integrated psychosocial, sleep, and nutritional support improves postoperative recovery, immune modulation, and survival after curative resection for low rectal cancer: a randomized controlled trial. Am. J. Surg. 256:116795. doi: 10.1016/j.amjsurg.2025.116795, 41475954

[ref36] Wang J. Q. Wang Z. X. Du C. C. Zhao T. Y. Song X. F. Yan YP . (2024). Impact of WeChat platform-based multidisciplinary intervention on self-management level and psychological status of patients with colostomy after low rectal cancer surgery. Oncol Prog. 22:1435–1439.

[ref37] Xi Y. Xu P. (2021). Global colorectal cancer burden in 2020 and projections to 2040. Transl. Oncol. 14:101174. doi: 10.1016/j.tranon.2021.101174, 34243011 PMC8273208

[ref38] Xia W. Liu T. Wu M. Luo Z. Guo D. Tian L. (2025). The trajectory of caregiver burden and the predictive role of sense of coherence among primary caregivers of colorectal cancer patients: a longitudinal study. BMC Nurs. 24:1202. doi: 10.1186/s12912-025-03807-1, 41013519 PMC12465131

[ref39] Yan J. J. Mou S. Y. Tan R. F. Wang X. H. (2015). An empirical study on the Chinese version of the low anterior resection syndrome score. Mil Nurs J. 32:12–15.

[ref40] You C. Xie G. Lin S. Li S. Jia M. Wu X. . (2024). Temporal relationship between symptom cluster and quality of life in rectal cancer patients after laparoscopic anus-preserving surgery. Sci. Rep. 14:32079. doi: 10.1038/s41598-024-83755-z, 39738816 PMC11685488

[ref41] Zarghani S. S. Gorgi K. Bananzadeh A. Safarpour A. R. Hosseini S. V. (2025). Effects of low anterior resection syndrome after colorectal cancer resections on health-related quality of life: a systematic review and meta-analysis. Tech. Coloproctol. 29:114. doi: 10.1007/S10151-025-03136-0 40347378 PMC12065725

[ref42] Zhao G. Li C. Li J. Balluz L. S. (2013). Physical activity, psychological distress, and receipt of mental healthcare services among cancer survivors. J. Cancer Surviv. 7, 131–139. doi: 10.1007/s11764-012-0254-6, 23184465 PMC11250998

[ref43] Zhou C. C. He J. J. Xu L. Z. Sun H. Zheng W. G. Wang X. Z. . (2009). The first application of the Kessler 10 Scale in the assessment of mental health among the elderly in China. Chin J Clin Psychol. 17:761–763.

[ref44] Zivanov C. N. Owoade I. A. Mann E. J. Adedeji O. E. Bassey G. I. Fayenuwo O. J. . (2025). Understanding colorectal cancer-related stigma and its impact on cancer survivors in South–West Nigeria: a qualitative study of patients, caregivers, and healthcare professionals. J. Cancer Surviv. doi: 10.1007/s11764-025-01953-8, 41432978

